# Silicone breast implant modification review: overcoming capsular contracture

**DOI:** 10.1186/s40824-018-0147-5

**Published:** 2018-12-20

**Authors:** Byung Ho Shin, Byung Hwi Kim, Sujin Kim, Kangwon Lee, Young Bin Choy, Chan Yeong Heo

**Affiliations:** 1 Department of Biomedical Engineering, Seoul National University College of Medicine, Seoul, 03080 Republic of Korea; 2 Department of Transdisciplinary Studies, Graduate School of Convergence Science and Technology, Seoul National University, Seoul, 08826 Republic of Korea; 3 Interdisciplinary Program for Bioengineering, College of Engineering, Seoul National University, Seoul, 08826 Republic of Korea; 4 Department of Plastic and Reconstructive Surgery, College of Medicine, Seoul National University, Seoul, 03080 Republic of Korea; 5 Department of Plastic and Reconstructive Surgery, Seoul National University Bundang Hospital, Seongnam, 13620 Republic of Korea; 6 Institute of Medical & Biological Engineering, Medical Research Center, Seoul National University, Seoul, 03080 Republic of Korea; 7 Advanced Institutes of Convergence Technology, Suwon, Gyeonggi-do 16229 South Korea

**Keywords:** Fibrosis, Inflammation, Silicone implant, Foreign body reaction, Capsular contracture, Modification

## Abstract

**Background:**

Silicone implants are biomaterials that are frequently used in the medical industry due to their physiological inertness and low toxicity. However, capsular contracture remains a concern in long-term transplantation. To date, several studies have been conducted to overcome this problem. This review summarizes and explores these trends.

**Main body:**

First, we examined the overall foreign body response from initial inflammation to fibrosis capsule formation in detail and introduced various studies to overcome capsular contracture. Secondly, we introduced that the main research approaches are to inhibit fibrosis with anti-inflammatory drugs or antibiotics, to control the topography of the surface of silicone implants, and to administer plasma treatment. Each study examined aspects of the various mechanisms by which capsular contracture could occur, and addressed the effects of inhibiting fibrosis.

**Conclusion:**

This review introduces various silicone surface modification methods to date and examines their limitations. This review will help identify new directions in inhibiting the fibrosis of silicone implants.

## Introduction

Silicone is the most common material used in medical devices that are inserted into the human body due to its physiological inertness, low toxicity, and antiadhesive properties. In particular, silicone implants are used in plastic surgery for breast augmentation and breast reconstruction [1]. According to the Plastic Surgery Statistics Report of 2017, 300,378 breast augmentation operations were performed—a 3% increase from 2016. Silicone implant surgeries are the most common type of cosmetic surgery cases. However, this surgery is often accompanied by capsular contracture (CC) with an incidence of approximately 10.6% [2, 3]. CC results from the immune response to a foreign body, causing pain and discomfort and resulting in the distortion of both the implant and the patient’s chest [4]. CC has long been studied; however, the precise mechanism by which it occurs has not yet been clarified. Bacterial contamination and the foreign body reaction (FBR) are known to be the main causes.

Implants are being improved to overcome these problems. The first improvement involves reducing bacterial contamination of the implant, and the second involves modifications to minimize the FBR. Whereas second-generation implants focused on functional and aesthetic improvements, in third- and fourth-generation implants, the shell was modified to reduce leakage of the gel filling agent and to increase the cohesion of the gel itself [5–10]. Through these improvements, an anatomical model was produced. When fifth-generation implants emerged, the CC incidence was reduced, and rupture of the silicone implants decreased. The safety of the implants also improved [8, 10]. The current sixth-generation implants focus on surface modifications that minimize the FBR. However, varying degrees of CC are still reported, depending on the topography of these surfaces [11, 12]. Previous studies have shown that more malignant CC occurs in smooth implants than in textured implants. A microtextured implant represents a compromise between smooth and textured implants.

In this review, we introduce silicone implant modification methods to reduce bacterial contamination and the FBR. In addition to physical surface modifications, we also introduce various chemical surface modifications of silicone implants currently under study.

## Mechanism of fibrosis

Fibrosis is a complicated process which is initiated by various reactions in vivo and progresses due to different reactions of various cellular factors. The most common fibrosis reaction is the FBR, which occurs when biomaterials are implanted. Fibrosis is an in vivo defense mechanism caused by infection, autoimmune factors, foreign material insertion, spontaneous factors, and cancer [13]. It occurs through a chain reaction of various factors—most frequently through a reaction with a foreign body—and its frequency has gradually increased with the increasing number of breast augmentation surgeries that introduce diverse biomaterials into the body [14, 15].

In this review, we focus on the occurrence of fibrosis via the implantation of biomaterials. The fibrotic response to biomaterials is caused by an immune reaction, and the overall 6-step reaction occurs through adaptive immune reaction over a certain period. Cellular activity is altered depending on the duration of the immune reaction, and the final effect of fibrosis occurs via changes in the expression pattern of the involved factors [16, 17]. The stages of fibrosis are as follows: 1. blood-biomaterial interaction, 2. provisional matrix formation, 3. acute inflammation, 4. chronic inflammation, 5. foreign body giant cell formation, and 6. fibrous capsule formation [4, 18].

### Blood-biomaterial interaction

The blood-biomaterial interaction is initialized by the leakage of blood at the wound site when an implant is inserted and results in the surface bonding of proteins in the blood. This protein surface adsorption differs depending on the surface characteristics of the implant [19]. In general, adsorption of the proteins in the blood occurs first, and subsequent binding of additional proteins occurs due to the presence of cells at the implantation site. The proteins thus induce cellular activity through the binding of cells present in vivo [20]. This step plays a major role in inhibiting the surface exposure of primary implants through the accumulation of proteins on the surface of the foreign material, which causes cellular activation through surface-adsorbed proteins. A protein layer with a thickness of 2–5 nm is formed on the surface of the biomaterials, which induces the formation of a provisional matrix through cellular activation and the additional adsorption of proteins [18, 21]. This effect is called the Vroman effect [22, 23]. At the beginning of this step, the albumin present in the blood accumulates on the surface, and over time, it is replaced by high-affinity proteins, such as fibrinogen, kininogen, fibronectin, and vitronectin.

### Provisional matrix formation

In the provisional matrix formation step, high-affinity proteins (i.e., fibrinogen, kininogen, fibronectin, and vitronectin) combine to form a provisional matrix [24, 25]. The fibrinogen present in the matrix is replaced with fibrin over time, although some fibrinogen remains for platelet binding and the cellular activity that occurs during the next phase [22, 26]. Additionally, the matrix formed during this step adsorbs various factors through the residual fibrinogen and thereby contains factors that activate cells, such as macrophages, during the next stage [20]. In this step, preparations for cellular activation and activity factors (von Willebrand factor) present in the blood are captured in the provisional matrix via alpha integrin. Based on this information, preparations for the progression of inflammation can be considered [4].

### Acute inflammation

Acute inflammation is an early stage of the cellular reaction during the development of fibrosis and is caused by multiple reactions of various inflammatory cells (i.e., neutrophils, eosinophils, basophils and others) [27, 28]. Acute inflammation occurs in a short time, ranging from a few hours to a few days, and is linked to chronic inflammation that occurs at the end of acute inflammation. Neutrophils and eosinophils (polymorphonuclear cells; PMNs) play a major role in this early inflammatory reaction and activate cells through the expression of various factors (i.e., TNF-α, interleukin family, IFN-γ) [27, 29]. Neutrophils induce inflammation through the secretion of several cytokines and induce fibrosis through the release of matrix metalloproteinases (MMPs), elastase and cathepsin [30]. Mast cells and PMN-derived cellular factors present at this step are also known to play a major role in fibrosis [31]. Histamine, IL-4, and IL-13 expressed by mast cells, as well as IL-8, monocyte chemoattractant protein-1 (MCP-1), and macrophage inflammatory protein-1ß (MIP1ß), increase the recruitment of leukocytes and the number of macrophages. Thus, acute inflammation progresses through a fibrotic reaction and leads to chronic inflammation [27].

### Chronic inflammation

Chronic inflammation occurs for 2–3 weeks, during which the cytokines and cells for fibrosis are recruited and activated. Lymphocyte and monocyte infiltration primarily occur, and throughout these phenomena, cells remain close to the biomaterials and secrete IL-4, IL-13, and other factors [32, 33]. These secreted factors affect macrophage activity and cause fusion into foreign body giant cells (FBGCs). In chronic inflammation, cytokines that induce collagen synthesis in the periphery of biomaterials are primarily expressed, rather than platelet-derived growth factor (PDGF), vascular endothelial growth factor (VEGF), or transforming growth factor beta (TGF- β) [34–36]. The MCP and MIP cytokine families are also expressed. During this period, macrophages play a major role. They are recruited by various factors and are expressed and activated during the fibrotic reaction. In chronic inflammation, granulation tissue forms around the biomaterials through the activity of the factors and cells described above [30, 37].

### Foreign body reaction

The FBR step is the stage during which the FBGCs formed during chronic inflammation generate fibrosis. It is also the step during which cells that play a major role in the synthesis of collagen, such as fibroblasts, myofibroblasts, and FBGCs, are activated. FBGCs are formed by the fusion of macrophages that are activated via specific cytokines, and when the implant is present, this formation is maintained for a longer period. These FBGCs remove foreign substances in vivo through phagocytosis and cause cell activation. FBGCs express CD11, CD45, and CD31 proteins as well as other receptors capable of binding to IL-1, IL-2, IL-4, and IL-8 on their surface membranes [4, 38]. Notably, the expression patterns of macrophages in vivo are distinct (M1 and M2 macrophages), and the expression patterns of various cytokines vary according to the phenotype of each macrophage. IL-10, TGF-β, and MCP-1 are expressed in the early phase of the FBR, as are IL-1α, IL-6, IL8 and TNF-α, which are proinflammatory cytokines [39, 40]. The expression of these factors modulates the activity of macrophages and FBGCs, determines the presence or absence of fibrosis at the capsule formation stage, and regulates fibrosis severity by changing the activity of the factors according to the characteristics of the biomaterials [38].

### Fibrous capsule formation

Finally, the fibrous capsule is formed through the several preceding steps, and fibrosis is terminated. In this step, collagen is synthesized in the peripheral region to isolate foreign materials in vivo, thereby stabilizing the biological reaction by reducing the stimulation of all biological reactions. At this stage, factors typically expressed by M2 macrophages play a major role in controlling collagen synthesis [39]. PDGF, VEGF, and TGF-β are typical factors that induce collagen synthesis and ECM remodeling by stimulating keratinocytes, fibroblasts, endothelial cells, thrombocytes, and adipocytes, which play a major role in collagen synthesis [31, 41]. Initially, collagen type III is synthesized to form a coarse matrix; over time, this matrix stabilizes and replaces collagen type I, resulting in complete isolation [42]. After this step, the in vivo reaction is stabilized, and the immune response by foreign materials in vivo is also stabilized. However, if isolation is not properly performed at this step, more severe fibrosis, such as scarring and CC, will occur.

## Surface modification

### Modification of surface topography

#### Smooth surface

The earliest form of silicone breast implant had a smooth surface and was made commercially available with little knowledge of the in vivo immune response. These smooth silicone implants continue to be used to the present day but are still reported to cause severe fibrosis. According to previous studies, smooth implants cause a reduced inflammatory reaction in in vivo implantation as well as reduced physical stimulation [43, 44]. Although smooth implants have been used consistently as implantable devices for breast reconstruction because of these advantages, their frequency of use is decreasing because the incidence of CC is greater with the use of smooth implants than other surface types. Furthermore, because of the inability to fix the smooth silicone breast implant by generating seroma in vivo [45], smooth silicone breast implants are no longer recommended [46, 47]. However, despite the drawbacks of smooth silicone breast implants, they are still being used due to their ability to create a perfect circular breast shape.

#### Textured surface

Implants with a textured surface represent an improvement over smooth-surfaced silicone breast implants: textured implants with a surface roughness of 100–300 μm avoid fibrosis. Textured implants have been developed through three generations of implant designs and continue to improve fibrosis inhibition as information about in vivo fibrosis is acquired [48]. In textured silicone breast implants, the capsule tissue of the collagen is constructed during in vivo implantation, which facilitates the fixing of the implant position in vivo*.* Because of this advantage, textured silicone breast implants are currently used in breast reconstruction surgery and are continuously being developed because they can be formed into various shapes by facile in vivo fixation. Droplet-shaped implants are available on the market today only with a textured surfaces. Textured silicone breast implants are known to cause less fibrosis in vivo, and the frequency of CC relative to smooth implants is 5–10% less [43]. Textured silicone breast implants are frequently used for various drug-loaded implants, which are being developed as the next generation of implants due to the ease of drug loading on the surface and sustained drug release from the surface in vivo. From this perspective, the development of functional silicone breast implants with various fibrosis-inhibiting drugs (tranilast, zafirlukast, montelukast, etc.) has been reported [49–51]. A recent study, however, has reported a strong association between these implants and anaplastic large cell lymphoma (ALCL), and they are therefore being replaced by the next generation of implants [52].

#### Micro/Nanotextured surface

Implants with micro- or nanotextured surfaces have been developed as next-generation implants. By controlling the roughness of the existing textured surface on the micro- or nanoscale, implants with characteristics of both smooth and textured surfaces can be developed [53]. Based on studies of commercial products, fibrosis occurs less frequently in the in vivo implantation of these latest implants. In general, micro- and nanotexturing of the surface of silicone breast implants produces a surface roughness of 10 to 100 μm, and fibrosis in vivo is reduced due to the surface topography of textured silicone breast implants. Additionally, the frequency of CC will eventually decrease with decreased of inflammation in the early inflammation phase compared to that in smooth-surfaced silicone breast implants. However, since micro and nanotextured surface implants have only recently been developed, clinical data are not sufficient and the stability of implant fixation remains uncertain.

### Antiadhesive modification

To avoid the FBR, silicone implant materials may be treated to obtain antiadhesion properties. There are some reports of CC suppression when such methods are used [54–57]. For example, a study by Park et al. investigated Guardix-SG, a biodegradable membrane that acts as an antiadhesion barrier [56]. Other conventional antiadhesion agents are composed of highly viscous components, and their physical properties are fixed. However, Guardix-SG, which is composed of alginate and poloxamer, changes its physical properties in gel form when the temperature rises. In a similar case, a study by Lew et al. showed that in rats, capsule thickness was significantly decreased in the experimental group compared to the control group when antiadhesion barrier solution (AABS) was used [57]. In this paper, hyaluronic acid (HA) was used as an AABS, and there was a reduction of approximately 53% in the capsule thickness compared to that of the saline-treated group. In the rat in vivo experiment, the AABS volume was equal to the volume of the implant, but a different volume could be expected to be effective in a clinical trial. If AABS is tested in clinical trials, the optimal concentration to cover the implant to inhibit CC will be found.

### Antibacterial modification

Several papers have shown that CC occurs due to bacterial presence as well as an FBR to silicone, and also confirmed that bacterial colonization is closely related to high-grade capsular contracture [58, 59]. For examples, In the patients who experienced CC, the presence of bacteria was confirmed by isolating the strains through vortexing and sonication after implant removal surgery. Furthermore, the serum hyaluronan level of the patient showed a statistically significant increase, with a high Baker grade in CC patients [58, 60]. Additionally, porcine in vivo studies that involve the inoculation of a human strain of *Staphylococcus epidermidis* on silicone implants show that the presence of bacteria has a significant impact on CC [61]. The exact mechanism by which the presence of biofilm affects CC is unknown, but research has shown that the incidence of CC can be lowered by using antibiotics and povidone-iodine irrigation to eliminate bacteria [62, 63]. In addition to these surgical treatments, several studies have reported that the introduction of antibacterial properties into the implant itself may lower the incidence of CC.

#### Plasma-assisted surface modification

Plasma is defined as an ionized gas that has the same amount of negatively and positively charged ions. Plasma treatment was proven to be a highly effective sterilization measure against *Escherichia coli, Staphylococcus* strains, *Pseudomonas aeruginosa* and *Bacillus strains* in several studies [64]. In contrast to autoclave sterilization, plasma sterilization has the advantage that the properties of the material can be preserved at high temperature and high pressure [65]. Additionally, because the residue after sterilization is nontoxic, the nonionized original gas does not pose any safety risks to researchers and users. Oxygen (O_2_) is the most commonly used gas. O, OH and OOH are created when oxygen is in a plasma state. OH is known to have the highest sterilizing power, and its sterilizing efficiency increases with O_2_ concentration [64, 66].

#### Modification of the hydrophilic surface by plasma treatment

Silicone breast implants are hydrophobic. Therefore, there is a material limitation that the implant itself cannot contain the hydrophilic antibiotic and povidone-iodine. However, when plasma treatment is performed, OH groups are present on the surface, and the silicone surface becomes hydrophilic. In the work of Barnea Y, et al., each group was irrigated after the plasma treatment or no treatment, and the sterilization efficiency was substantially improved in the plasma-treated implants [62, 67].

Silicone shells with the ability to absorb water after plasma treatment exhibit prolonged antibacterial properties. In the case of non-plasma-treated silicone implants, fluorescence microscopy showed that the surface of the shell disc was covered with a layer of *P. aeruginosa*. Treatment with gentamicin removed a small number of bacteria, and the surface was slightly improved. However, in the plasma-treated group, complete eradication of the bacterial layer was observed.

#### Direct treatment with antibiotics

The inoculation of smooth silicone implants with *S. epidermidis* extracted from actual CC patients resulted in a spherical formation with a high Baker grade; however, the incidence of CC significantly decreased when patients were treated with an antibiotic-impregnated mesh [61]. In addition, in a study by Jacombs A, et al., SEM analysis of an implant removed 16 weeks after transplantation demonstrated that the antibiotic-treated group formed less biofilm.

This finding suggests that the inhibition of bacterial adhesion during breast implant insertion can be used as a strategy to inhibit biofilm formation. The antibiotic-impregnated mesh showed a considerable influence on the Baker grade. In addition, the tonometry data showed that the antibiotic-impregnated mesh had a large surface area. This finding implies that even smaller volumes of silicone implants can be as safe and effective as existing implants [61].

### Surface modification using antifibrosis drugs

#### Triamcinolone

Steroids are widely used to promote anti-inflammatory activity. Glucocorticoids are known to inhibit inflammatory cytokines, such as TNF-α and IL--1β, at the gene level [68–70]. In addition to direct action, the downregulation of chemoattractants and adhesion molecules that promote the invasion of inflammatory cells also leads to overall anti-inflammatory effects [71]. In addition, exposure to this drug can be expected to inhibit direct or indirect fibrosis by blocking the inflow of PMNs and monocytes and reducing TGF-β production. The implant was coated to perform sustained release of the drug and thus to effectively suppress fibrosis and CC around the silicone implant. Triamcinolone is an FDA-approved drug that is usually not continuously administered but instead is often administered via a local shot. However, in the case of glucocorticoids, continuous exposure may cause various side effects. Typical symptoms are skin thinning and muscle loss. Therefore, it is very important to treat the patient with the correct dose. Notably, these side effects were observed in the experimental group at a high concentration, but there were no side effects within the therapeutic window; rather, decreased collagen density and the inhibition of various fibrosis-related cytokines were observed [72]. Inflammation levels due to the FBR were also significantly lower than those in the controls, especially for early inflammation. Notably, the amount used in the in vivo model was approximately 2000-fold lower than the dose applied in clinical practice, and this low concentration proved to be sufficient for local sustained delivery [72]. However, adverse effects remain an issue.

#### Tranilast

When selecting a drug, it is very important to select the pathway intended to block fibrosis. Tranilast is a drug that targets TGF-β by directly blocking the secretion of TGF-ß and inhibiting the expression of its receptor. This effect inhibits the phosphorylation of the Smad pathway, directly or indirectly blocking TGF-β family signaling [73–75]. Tranilast is mainly used to treat asthma, keloid scars, and hypertrophic scars, but it is not used to suppress CC. However, its ability to inhibit fibroblast and collagen production was observed and applied to silicone implants.

Experiments on its efficacy in an in vivo rat model showed a noticeable decrease in capsule thickness and collagen density in drug-treated implants 12 weeks after transplantation. Although the drug was released steadily, most of the drug was released during the first 5 days. However, TGF-ß expression was still decreased after 4 weeks. These results suggest that inhibition of capsule formation by inhibiting macrophage and fibroblast infiltration into the silicone implant site can be inhibited only by early TGF-beta inhibition [50].

#### Montelukast and zafirlukast

Montelukast and zafirlukast are drugs used in clinical practice as inhibitors of cysteinyl leukotrienes (CysLTs) [76–78]. When silicone implants are implanted and chronic inflammation occurs, CysLTs cause the migration of fibroblasts to the surface of the implants and promote fibroblast differentiation into myofibroblasts. When fibroblasts become myofibroblasts, alpha smooth muscle actin (α-SMA) expression increases with collagen production, and the ability to contract [79–81] is strengthened, which leads to CC. Montelukast is a drug that is known to inhibit CysLT production [82, 83]. It is a specific leukotriene receptor antagonist that specifically inhibits the production of leukotriene D4 (LTD4). Montelukast is known to bind to the type 1 CysLT receptor (CysLT1) present in the cell membrane of PMNs [82–84]. It is also suitable for breast implants because of its ability to reduce fibroblasts and myofibroblasts and to inhibit collagen production.

When montelukast was coated on the implants, the production of CysLTs and the number of fibroblasts decreased, as expected, in the in vivo rat model, and this effect was confirmed to be stronger when the drug was delivered for a longer period with poly(lactic-co-glycolic acid) (PLGA). This finding confirms that the inhibition of TGF-β expression and the number of myofibroblasts can be reduced by inhibiting CysLT production alone. However, macrophages were not effectively reduced, likely because CysLT receptors were more abundant in fibroblasts than in macrophages [85].

Likewise, zafirlukast is also a leukotriene receptor antagonist used orally to treat asthma. Similarly, the ability to inhibit eosinophilic recruiting and inhibit the ability of smooth muscle can be expected to inhibit fibrosis when applied to CC [86]. The work of A. Spano et al. showed effective suppression of capsule formation on both sides of the silicone disk. The inflammatory response control group was surrounded by granulocytes and a large number of eosinophils, while the inflammatory response was also lower in the drug treatment group [49].

#### Halofuginone

Halofuginone is a substance that interferes with Smad3 phosphorylation in the TGF-β signaling pathway in a manner similar to that of tranilast. Halofuginone has been used as an inhibitor of various types of fibrosis by inhibiting collagen I production [87–89]. However, because the systemic use of drugs can have many side effects, it may be beneficial to apply them to medical devices that can be delivered locally, such as silicone implants. Therefore, the main purpose of the study by McGaha et al. was to determine whether coating an implant with halofuginone could inhibit CC from forming at the foreign body [90]. No side effects were found when the drug was delivered to the local site. After 3 months, it was confirmed that in the drug treatment group, the inflammatory cell, collagen density, capsule thickness, and TGF-β levels were significantly reduced; collagen type 1 and 3 levels were also reduced [90].

## Conclusion

**Fig. 1:**
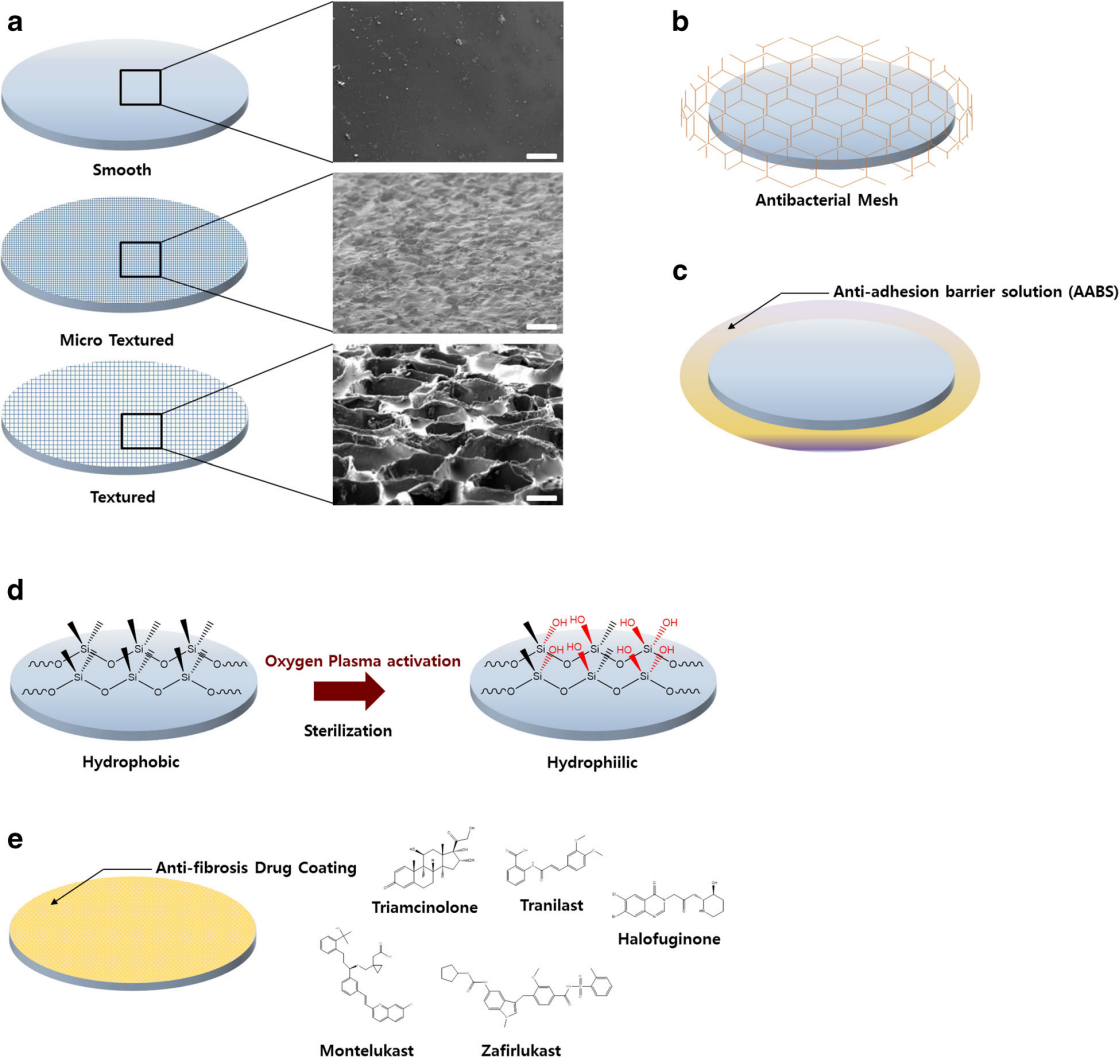
Overview of different PDMS breast implant surface modifications. **a** SEM images and scheme of PDMS breast implants with smooth, microtextured, and textured surface morphology. The scale bar is 100 μm. The schematic depicts (**b**) antibacterial mesh, (**c**) antiadhesion barrier solution (AABS), (**d**) oxygen plasma modification to change hydrophobicity to hydrophilicity, and (**e**) antifibrosis drugs coated on the PDMS breast implant surface

This work examined the overall research progress related to the physical and chemical surface modification of silicone breast implants to inhibit capsule formation by fibrosis (Fig. 1, Table 1). We primarily discussed microscale inflammation and antibiofilm effects. However, in the future, nanotechnology will lead to further research, and the molecular mechanism of fibrosis, which has not yet been solved, will be analyzed using molecular and genetic analyses. These studies are not limited to silicone implants but have various potential applications, such as in pacemakers, joint replacements, and esophageal stents, and can provide insights into the biointegration of medical devices into the human body. Until now, researchers have found it difficult to translate academic results to industrial products or have stopped research in this area. However, further studies that could be practically applied to the real market should be carried out.

**Table 1:** Overview of the characteristics of PDMS breast implant surface modification methods

Modification Methods	Type	Characteristics
Roughness Modification	Smooth	Severe fibrosis, High CC incidence rate
	Textured	Surface roughness of 100–300 μm, Fixing of the implant position, Known to cause less fibrosis, ALCL
	Micro Textued	Surface roughness of 10 to 100 μm, Low CC incidence rate
Anti- adhesion barrier solution (AABS) treatment	•	Reducing inflammation and fibrosis formation
Anti-bacterial	•	Lower the incidence of CC
Plasma treatment	•	Increased wettability, Easy to functionalize
Anti-fibrosis drug coating	Triamcinolone	Glucocorticoids, Effectively suppress fibrosis and CC, Cause side effects
	Tranilast	Drug that targets TGF-β, Suppress fibrosis and CC
	Montelukast/zafirlukast	Inhibitors of Cysteinyl leukotriene (CysLTs), Suppress fibrosis and CC
	Halofuginone	Interferes with Smad3 phosphorylation in the TGF-β signaling pathway, Cause side effects, Suppress fibrosis and CC

## Abbreviations

AABS: Antiadhesion barrier solution
ALCL: Anaplastic large cell lymphoma
CC: Capsular contracture
CD: Cluster of differentiation
CysLT: Cysteinyl leukotriene
ECM: Extracellular matrix
FBGCs: Foreign body giant cells
FBR: Foreign body reaction
FDA: Food and Drug Administration
HA: Hyaluronic acid;
IFN-γ: Interferon-gamma receptor
IL: Interleukin
LTD4: Leukotriene D4
MCP-1: Monocyte chemoattractant protein-1
MIP1ß: Macrophage inflammatory protein-1ß
MMPs: Metalloproteinases
PDGF: Platelet-derived growth factor
PLGA: Poly(lactic-co-glycolic acid)
PMNs: Polymorphonuclear cells
TGF-β: Transforming growth factor beta
TNF-α: Tumor necrosis factor alpha
VEGF: Vascular endothelial growth factor
α-SMA: Alpha smooth muscle actin
